# Gut microbiota profiling in Norwegian weaner pigs reveals potentially beneficial effects of a high-fiber rapeseed diet

**DOI:** 10.1371/journal.pone.0209439

**Published:** 2018-12-20

**Authors:** Özgün Candan Onarman Umu, Aud Kari Fauske, Caroline Piercey Åkesson, Marta Pérez de Nanclares, Randi Sørby, Charles McLean Press, Margareth Øverland, Henning Sørum

**Affiliations:** 1 Department of Food Safety and Infection Biology, Faculty of Veterinary Medicine, Norwegian University of Life Sciences, Oslo, Norway; 2 Department of Basic Sciences and Aquatic Medicine, Faculty of Veterinary Medicine, Norwegian University of Life Sciences, Oslo, Norway; 3 Department of Animal and Aquacultural Sciences, Faculty of Biosciences, Norwegian University of Life Sciences, Ås, Norway; National Institute for Agronomic Research, FRANCE

## Abstract

Rapeseed meal (RSM) is an alternative feed ingredient to soybean meal (SBM) in pig diets. However, knowledge on the effect of RSM on gut health, especially in relation to changes in gut microbiota is still limited. In our study, Norwegian Landrace weaner pigs were fed with either a control diet (CON) based on wheat, barley and SBM, or a high-fiber experimental diet where SBM was replaced by RSM (RSF). We found no large differences in the gut microbiota of pigs fed the two diets, suggesting that RSF does not disturb the gut microbiota and the normal gut function. The relative abundance of SCFA-producing phylotypes and colon-health related phylotypes increased in the large intestine of RSF-fed pigs. Among them, *Lachnospira* and *Coprococcus* were negatively associated with the presence of neutrophils in the colon wall. The higher abundance of these bacteria in colon of RSF pigs may suggest an anti-inflammatory stimulus effect of the RSF diet. The gut microbiota of RSF-fed pigs was relatively unaltered following episodes of diarrhea suggesting that the RSF diet may promote robustness in weaner pigs and reduce the risk of dysbiosis.

## Introduction

European pig production is highly dependent on imported protein-rich feed ingredients, such as soybean meal (SBM) [1]. The global agricultural market for SBM experiences price volatility and rapid changes in the availability. The European animal industry needs locally produced alternative protein feed ingredients to reduce its reliance on imports and to increase its self-sufficiency in animal production.

Rapeseed meal (RSM) is a co-product derived from the production of rapeseed oil and is a viable alternative to SBM in pig feeds owing to its high protein content [2]. It also contains a notable amount of dietary fiber including predominantly insoluble fiber, which helps maintain normal gut function [3]. On the other hand, there are some limiting factors for the use of RSM in pig diet. Anti-nutritional factors in RSM, such as glucosinolates, erucic acid, sinapines, tannins and phytic acid may lead to reduced feed intake, reduced growth performance and metabolic disorders in pigs [4,5]. Moreover, the dietary fiber content of rapeseed co-products in pig diet can be associated with impaired nutrient utilization and reduced net energy values [6]. However, despite of these limiting factors, RSM potentially has beneficial effects via fermentation of its dietary fiber content by the gut microbiota [3].

The gut microbiota has an important role in the fermentation of dietary fibers, as well as other dietary components, that are able to reach the large intestine. As a result, the microbiota modulates energy uptake from feed, growth and development of the body, and immune system of the host [7]. Even though RSM can serve as a good alternative protein source in pig diets and gut microbiota is an important factor in the physiological response of the host to the diet, a limited number of studies exists on the effects of RSM on gut microbiota in pigs. Replacing SBM with RSM in diets for growing-finishing pigs has shown to influence some bacterial groups in the gut [8] resulting in higher levels of *Lactobacillus* spp. and lower levels of *Enterococcus* spp. in caecum, while the family Enterobacteriaceae increased and *Clostridium perfringens* decreased in abundance in mid-colon. However, the study was limited to a few bacterial groups; and therefore, more detailed and comprehensive studies are required to get a more in-depth information about the impact on how feeding RSF will affect the gut health in relation to changes in the gut microbiota and the host-microbiota interaction.

In this study, the effects of a RSM-based diet on the gut microbiota of growing piglets were assessed by performing both culturing of specific bacterial groups and 16S rRNA gene sequencing to profile microbiota. Moreover, several host physiological parameters were analyzed in this study and in parallel studies of the same experiment. Correlation analyses were performed to investigate the associations between the gut microbiota and host physiological parameters.

## Materials and methods

### Animals and housing

The study was performed with 40 Norwegian Landrace castrated male weaner pigs (average age of 56 days) at the experimental farm of the Norwegian University of Life Sciences (NMBU), Aas, Norway. Each group of 20 pigs was fed either a control diet (CON) based on wheat, barley and SBM or an experimental rapeseed diet (RSF) where wheat and SBM were replaced by a mixture of 20% coarse RSM and 4% pure rapeseed hulls. Detailed experimental design, diet formulations and tissue and digesta sampling are described in Perez de Nanclares et al. [9]. Briefly, pigs were fed twice a day with the total amount of feed corresponding to 3.5% of their body weight per day, and their access to water was *ad libitum*. The experiment was conducted in two periods with 10 pigs per treatment per period and each period lasted for 3 weeks. After 3 weeks, all the pigs were euthanized and digesta samples from the distal ileum, the apex of caecum and the apex of the spiral colon were collected and kept at -80°C until the analysis. The research protocol was reviewed and approved by the Norwegian Food Safety Authority.

### Cultivation of bacteria

For the microbiological examination, 0.5 g digesta samples from ileum, caecum and colon of each pig were diluted in 4.5 ml 0.9% saline solution at collection, and subsequently serially diluted. Different selective agars (Oxoid, Cambridge, UK) were used for each bacterial group and 0.1 ml of the diluted digesta samples were plated. Lactic acid bacteria (LAB) were aerobically grown on de Man, Rogosa and Sharpe (MRS) agar at 35°C for 48 h. *Enterococcus* spp. were grown on Slanetz and Bartley agar aerobically at 35°C for 48 h. Coliforms were grown on MacConkey agar aerobically at 35°C for 24 h. *C*. *perfringens* was grown on Tryptose Sulfite Cycloserine (TSC) agar anaerobically at 35°C for 48 h. The following dilutions were used to plate the respective bacterial groups: 10^−6^, 10^−7^ and 10^−8^ for LAB; 10^−4^, 10^−5^ and 10^−6^ for *Enterococcus* spp.; 10^−3^, 10^−4^ and 10^−5^ for coliforms; 10^−3^, 10^−4^ and 10^−5^ for *C*. *perfringens*. The results were reported as log10 of colony-forming units (CFU) per gram digesta.

### DNA extraction and 16S rRNA gene sequencing

DNA extraction from the digesta samples was carried out using QIAamp DNA Stool Mini Kit (Qiagen, GmbH, Hilden, Germany) protocol of the QIAcube system (Qiagen, GmbH, Hilden, Germany). The kit protocol was followed after an additional step of bead beating at 30 Hz for 2 min using the TissueLyser system (Qiagen Retsch GmbH, Hannover, Germany). For bead beating, zirconia/glass beads (diameter, 0.1 mm, Carl Roth, Karlsruhe, Germany) were used.

The extracted DNA from the digesta samples was measured by Qubit® fluorometer using dsDNA BR or HS Assay Kits (Invitrogen, Eugene, OR, USA), and sent to GATC Biotech AG (Konstanz, Germany) for library preparation and sequencing of the 16S rRNA gene for microbiota profiling. The V1-V3 variable region of bacterial 16S rRNA gene was amplified using 27F (AGAGTTTGATCCTGGCTCAG) and 534R (ATTACCGCGGCTGCTGG) primers with output of 2 x 300 base-pair paired-end reads.

The fastq files have been deposited in the SRA (Bioproject ID: PRJNA427098 and Accession number: SRP127551).

### Analysis of 16S rRNA gene sequencing data

The paired-end, de-multiplexed Illumina reads were processed using the UPARSE pipeline [10] implemented in USEARCH [11] (version 9.2.64). Paired-ends were merged and quality filtering was applied using maximum expected error (maxee) value of 1.0. Sequences were dereplicated, and singletons were discarded. Sequences were clustered into operational taxonomic units (OTUs) using 97% sequence identity threshold, chimeric sequences were filtered from clustered OTUs using UCHIME [12] and an OTU table was created. OTUs were processed further using the Quantitative Insights Into Microbial Ecology (QIIME) version 1.9.1. The representative OTUs were picked and aligned against the Greengenes core set database [13] using PyNAST [14] with the default minimum identity of 75%. Taxonomy was assigned to aligned sequences using The Ribosomal Database Project (RDP) classifier program [15] with a confidence of 0.8. The OTUs with a fraction of total sequence count lower than 0.01 were filtered out from the OTU table. The OTU table was sub-sampled to normalize the sequence number among samples based on the sample with lowest number of sequences. A phylogenetic tree was built using Fast Tree [16] from aligned sequences after the filtration step to remove highly variable regions and positions that were all gaps. This tree was used to calculate alpha and beta diversities. Shannon indexes were calculated as the alpha diversity index. Unweighted UniFrac distance metrics [17] were generated and the principle coordinate analysis (PCoA) was used to visualize the metrics.

### Histopathology and immunohistochemistry

As reported in Perez de Nanclares et al. [9], a semi-quantitative analysis for the presence of IEL and infiltration of leukocytes (i.e., lymphocytes, plasma cells, eosinophils, neutrophils and macrophages) was performed on formalin-fixed, HE stained tissues from the colon of the pigs. The results for each pig were recorded as normal (0), mild (1), moderate (2) and severe (3) changes and presented individually. These data were used for the comparative analysis of the dietary treatment groups and the correlation analysis with the gut microbiota data.

Immunohistochemistry was performed on formalin-fixed paraffin embedded tissues from the colon of pigs to detect the presence of proliferating cells. Briefly, sections were cut at 4 μm and deparaffinized. Sections were autoclaved at 121°C in 0.01M citrate buffer for 15 minutes for antigen retrieval, and endogenous peroxidase was inhibited with 3.0% H_2_O_2_. After blocking with normal goat serum, the polyclonal Ki67 antibody (Abcam, Cat. no. ab15580) diluted 1:1000 was incubated one hour at room temperature, and biotinylated goat anti-rabbit secondary antibody (Vector Laboratories, Cat. no. BA-1000) diluted 1:50 was incubated 30 minutes at room temperature. Reagents from the Vectastaom Elite-HRP kit (Vector Laboratories, Cat.no. PK-6100) was applied for signal amplification, and immunoreactivity was detected using ImmPact AEC peroxidase substrate (Vector Laboratories, Cat.no. SK-4205), and finally slides were counterstained with hematoxylin.

A morphometric analysis was performed to estimate the presence of Ki-67+ cells in the colonic crypt epithelium using ImageJ software (National Institutes of Health, USA). Briefly, after calibration, crypt depth was measured in μm with the segmented line tool in all well-oriented crypts in one colonic section from each pig. In the same crypts, all Ki67+ cells were counted using the multipoint tool. The number of available well-oriented crypts ranged from 3 to 36 (except one pig that was excluded due to lack of well-oriented crypts). Mean crypt depth and mean number of Ki67+ cells per crypt were calculated.

### Digestibility and metabolites

Apparent ileal (AID) and total tract (ATTD) digestibilities of acid detergent fiber (ADF), neutral detergent fiber (NDF), crude protein (CP), dry matter (DM) and gross energy (GE) of all pigs were analyzed previously as described by Pérez de Nanclares et al. [9]. Briefly, it was shown that the RSF lowered apparent ileal digestibility and apparent total tract digestibility of ADF, NDF, CP, DM and GE. The digestibility data reported in Perez de Nanclares et al. [9] has been used for a correlation analysis with the gut microbiota. A correlation analysis was performed between the bacterial genera in ileum and AID, and bacterial genera in colon and ATTD because colon is the most representative part of the gastrointestinal tract in terms of microbiota for the feces.

A metabolomics analysis was performed on the digesta samples from the pigs in the present study using liquid chromatography-mass spectrometry (LC-MS) by Chen et al. [18].

### Statistics

The statistical analyses of microbiota and histology data and the correlation analyses were performed using the JMP Pro software (version 13, SAS Institute Inc.). Normality of the data was tested using the Shapiro-Wilk test [19]. Alpha diversity indices and the data from culturing were found to be normally distributed, and the effects of diets and the different periods were tested using student’s t-test and one-way ANOVA followed by post-hoc analyses with Tukey HSD test, respectively. The relative abundance data of the phylotypes from sequencing were found not to be normally distributed; therefore, the significance testing was performed using the non-parametric Kruskal–Wallis test with Dunn’s multiple comparison post-hoc test. Pearson correlations between the sequencing data and the histology, the digestibility and the metabolomics data were calculated, and heatmaps were plotted for metabolomics and bacteria correlations.

Proliferative acitivity in colon mucosa, mean crypt depth and mean number of Ki-67+ cells per crypt were compared between dietary treatments using student’s t-test, while non-parametric Wilcoxon signed-rank test was used for infiltration of leukocytes semi-quantitative data.

## Results

### The gut microbiota diversity and composition

The bacterial community structures in ileum, caecum and colon of pigs were assessed using 16S rRNA gene analysis. The average read count after quality filtering was 738,282 reads per sample and clustering analysis resulted in 2,025 OTUs in total at the 97% identity threshold.

**Fig 1:**
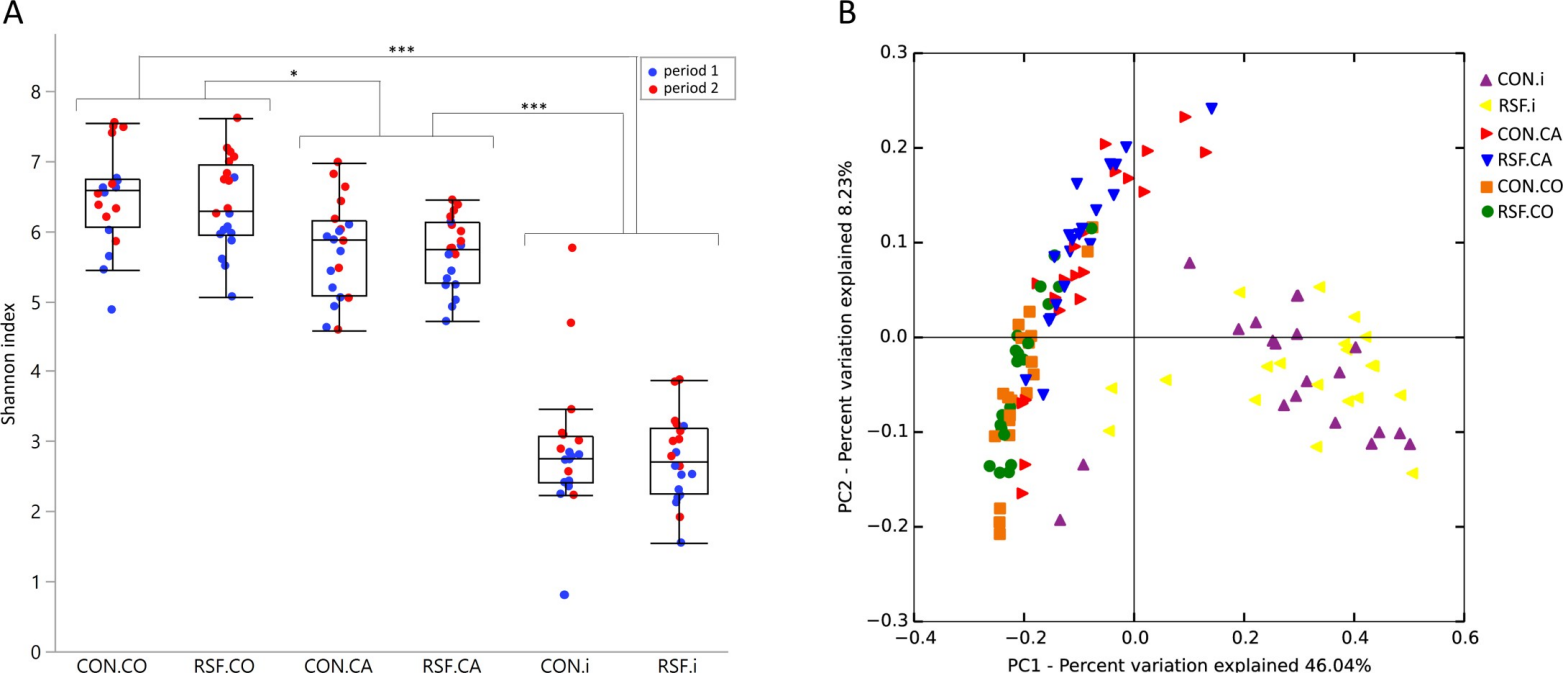
Gut community diversity and composition. (A) Bacterial diversity was represented by Shannon index. Each bar represents the samples from different gut locations of the pigs fed with either CON diet or RSF diet. Blue and red colored dots represent samples from experimental period 1 and 2 respectively. (B) Principle coordinate analysis (PCoA) plot generated based on the calculated distances in an unweighted UniFrac matrix. Samples were grouped by color and shape in terms of diet group and gut location of the pigs. CO, colon; CA, caecum; i, ileum.

Alpha diversity analysis was performed to determine the effect of the diets on the gut microbiota diversity. The diversity of bacterial OTUs (at the 97% identity) in the three sampled locations of the gastrointestinal tract, ileum, caecum and colon, was presented using Shannon index for both of the diet groups, indicating the samples from different experimental periods (Fig 1A). In general, there was no significant difference in gut microbiota diversity between the diets or the experimental periods, except for a higher diversity in colon of RSF pigs in the second experimental period compared with the first period (*P* < 0.05). However, the diversity was significantly different between the gut locations with the highest diversity in colon and the lowest in ileum of the pigs (Fig 1A), as expected due to the increasing bacterial load from the small intestine to the large intestine.

The distribution of the microbiota compositions in ileum, caecum and colon of the individual animals was consistent with the diversity results (Fig 1B). A separation appeared between the samples from different locations of the gut in terms of their microbiota compositions, which was most pronounced for the ileum samples. The samples from the pigs fed with different diets did not diverge from each other within each gut location.

### Taxonomic affiliations

The caecal and colonic microbiota of the pigs were dominated by the phyla Bacteroidetes (63.8% in caecum of the CON pigs and 63.1% in caecum of the RSF pigs; 63.6% in colon of the CON pigs and 63.7% in colon of the RSF pigs) and Firmicutes (29.4% in caecum of the CON pigs and 31.8% in caecum of the RSF pigs; 30.8% in colon of the CON pigs and 31.5% in colon of the RSF pigs). Other notable phyla present in caecum and colon included Proteobacteria, Actinobacteria and Spirochaetes. The most abundant phylum in ileum of the pigs was Firmicutes (93.8% in the CON pigs and 96.1% in the RSF pigs), and other abundant bacterial phyla were Bacteroidetes (3.4% in the CON pigs and 1.9% in the RSF pigs) and Proteobacteria (2.2% in the CON pigs and 1.4% in the RSF pigs). The only phylum that was significantly affected by the diet was Actinobacteria in caecum with higher abundance in the RSF pigs compared with the CON pigs (*P* < 0.05).

**Fig 2:**
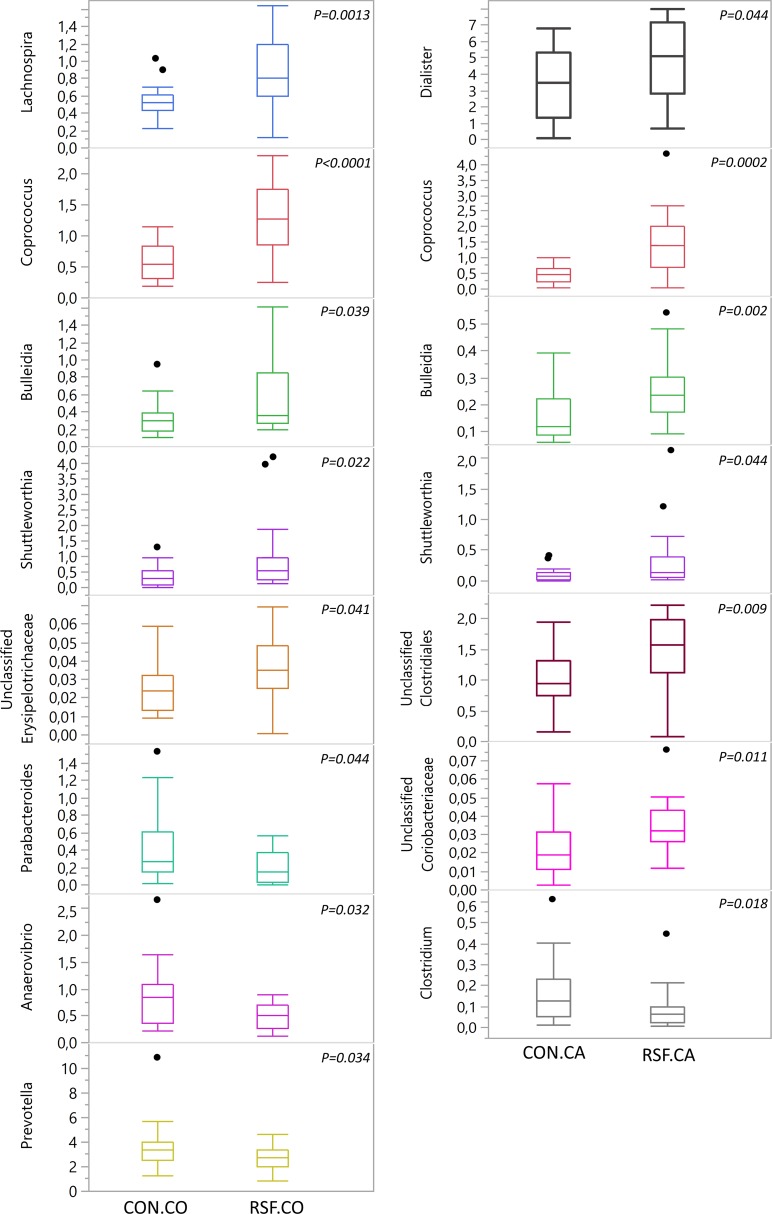
Significant changes in relative abundance of bacterial groups at genus level. CON.CO, in the colon of CON pigs; RSF.CO, in the colon of RSF pigs; CON.CA, in the caecum of CON pigs; RSF.CA, in the caecum of RSF pigs. Significance degree of difference between the diets is shown for the bacterial groups indicating *P*-value on each graph.

The effect of diets on relative abundance of bacteria was more pronounced at genus level in caecum and colon (Fig 2). Genera affiliated to the Firmicutes phylum such as *Lachnospira* (*P* < 0.01), *Coprococcus* (*P* < 0.0001), *Bulleidia* (*P* < 0.05), *Shuttleworthia* (*P* < 0.05) and unclassified Erysipelotrichaceae (*P* < 0.05) had a higher relative abundance in colon of the RSF pigs, while the relative abundance of *Anaerovibrio* decreased (*P* < 0.05). On the other hand, genera from the Bacteroidetes phylum such as *Parabacteroides* (*P* < 0.05) and *Prevotella* (affiliated to the Paraprevotellaceae family) (*P* < 0.05), were significantly less abundant in colon of the RSF pigs. In caecum, some of the same genera as in colon were more abundant in the RSF pigs; these included *Coprococcus* (*P* < 0.001), *Bulleidia* (*P* < 0.01) and *Shuttleworthia* (*P* < 0.05). In addition, higher abundances of other genera affiliated to the Firmicutes i.e. *Dialister* (*P* < 0.05), unclassified Clostridiales (*P* < 0.01) as well as unclassified Coriobacteriaceae (*P* < 0.05) from the Actinobacteria phylum were observed in caecum of the RSF pigs. The abundance of the *Clostridium* population was lower in the caecum of RSF pigs (*P* < 0.05).

Culturing of specific bacterial groups revealed that the abundance of *C*. *perfringens* was significantly changed by the diet, and was lower in caecum and colon of the RSF pigs compared with the CON pigs (*P* < 0.05) (Table 1). The decrease in *C*. *perfringens* in caecum of the RSF pigs was consistent with the decrease in *Clostridium* genus observed from the sequencing data, which indicates that the lower abundance of *Clostridium* in caecum was a consequence of the decrease in abundance of the *C*. *perfringens* species. On the other hand, other cultured bacterial groups were not found to be significantly different, which is consistent with the sequencing data (Table 1). There was no difference in the abundance of the cultured bacterial groups between the experimental periods in any of the locations of gut.

**Table 1:** Effects of control diet (CON) and rapeseed diet (RSF) on the cultured bacterial groups.

	Log CFU/g digesta (Mean ± SEM)		
Bacterial group	CON	RSF	*P*-value
Ileum			
Lactic acid bacteria	9,09 ± 0,17	8,70 ± 0,19	0,070
*Enterococcus*	5,22 ± 0,19	5,63 ± 0,20	0,928
Coliforms	6,70 ± 0,18	6,52 ± 0,23	0,263
*Clostridium perfringens*	4,27 ± 0,17	4,37 ± 0,40	0,606
Caecum			
Lactic acid bacteria	9,23 ± 0,11	9,08 ± 0,14	0,214
*Enterococcus*	5,68 ± 0,15	5,56 ± 0,18	0,299
Coliforms	7,22 ± 0,15	7,13 ± 0,15	0,331
*Clostridium perfringens*	7,23 ± 0,24	6,68 ± 0,19	0,045
Colon			
Lactic acid bacteria	9,42 ± 0,10	9,30 ± 0,11	0,223
*Enterococcus*	6,06 ± 0,16	5,85 ± 0,17	0,186
Coliforms	7,42 ± 0,15	7,12 ± 0,14	0,069
*Clostridium perfringens*	7,81 ± 0,16	7,36 ± 0,20	0,048

### Effects of the diets on the gut microbiota of the pigs that experienced diarrhea

Some of the animals from both of the dietary treatment groups (5 CON and 5 RSF pigs) showed signs of diarrhea in the second experimental period (S2 Table). These pigs were immediately treated with probiotic (ZooLac Propaste; VESO AS, Oslo, Norway) following veterinary recommendations, and all the pigs recovered within 1–5 days.

In terms of diversity and composition of the gut microbiota in ileum, three individual pigs that experienced diarrhea separated from the other pigs. Two individuals in the CON group (pig no. 36 and 38) and one in the RSF group (pig no. 37) had higher microbiota diversity in ileum. Moreover, the bacterial compositions in ileum of these pigs were more similar to the compositions in colon and caecum than in ileum of other pigs. These individual pigs had a much higher relative abundance of Bacteroidetes (pig no. 36, 35.8%; pig no. 38, 28% and pig no. 37, 13%) and Proteobacteria (pig no. 36, 6.5%; pig no. 38, 32% and pig no. 37, 20%) in ileum than the other pigs. This diversity may indicate a reflux of intestinal content and a bacterial translocation from caecum and/or colon to the ileum, resulting from the diarrhea.

**Fig 3:**
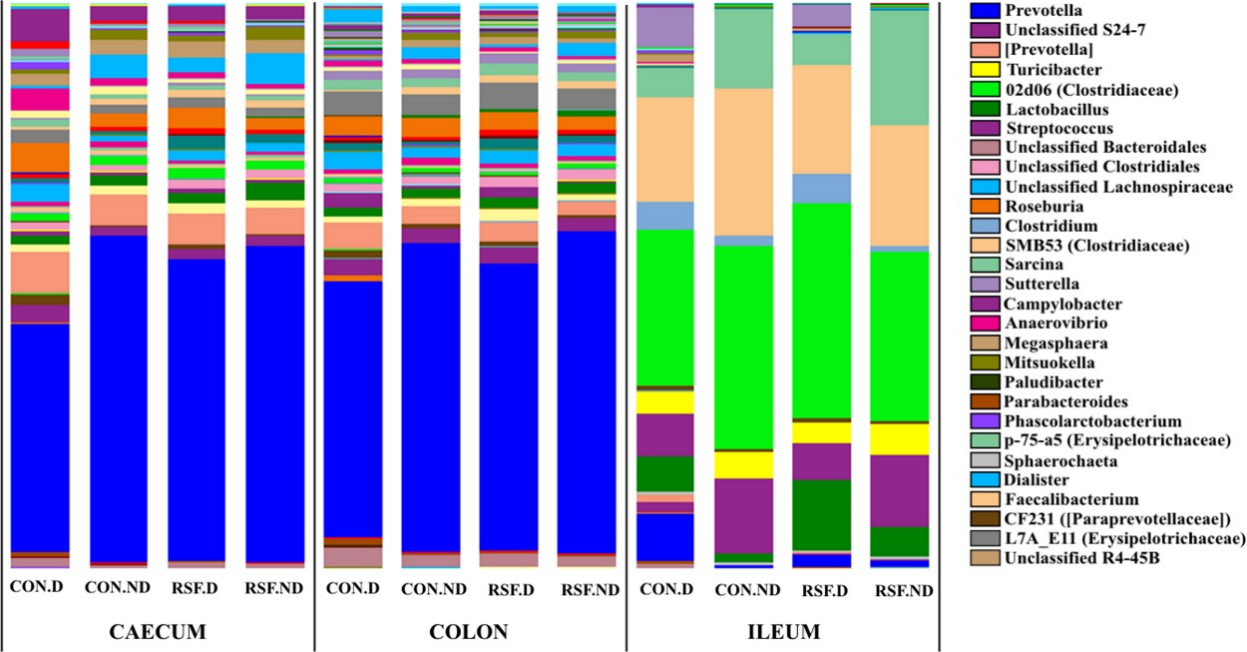
Bacteria in different gut locations of the pigs grouped depending on the incidence of diarrhea. Different colored bars represent different genera with size showing relative abundance of this genus to the total bacterial abundance. CON.D, CON diet fed pigs experienced diarrhea; CON.ND, CON diet fed pigs with no diarrhea; RSF.D, RSF diet fed pigs experienced diarrhea; RSF.ND, RSF diet fed pigs with no diarrhea.

The relative abundance of some bacterial phylotypes at genus level varied in the CON pigs that experienced diarrhea compared with the other CON pigs (Fig 3). In colon, *Paludibacter* (*P* < 0.05), *Parabacteroides* (*P* < 0.05), *Phascolarctobacterium* (*P* < 0.01), p-75-a5 (affiliated to Erysipelotrichaceae) (*P* < 0.05) and *Sphaerochaeta* (*P* < 0.05) were observed to be significantly higher in relative abundance in CON diarrhea-pigs, while some other groups, *Dialister* (*P* < 0.01), *Misuokella* (*P* < 0.01) and *Faecalibacterium* (strong tendency, *P* = 0.05) were lower in abundance. In caecum, some bacterial populations, including these genera, also varied in relative abundances in pigs that had experienced diarrhea. The bacterial groups with higher relative abundance in caecum of CON diarrhea pigs included *Paludibacter* (*P* < 0.01), *Parabacteroides* (*P* < 0.05), *Phascolarctobacterium* (*P* < 0.01), CF231 (affiliated to Paraprevotellaceae) (*P* < 0.05), *Anaerovibrio* (*P* < 0.05), L7A_E11 (affiliated to Erysipelotrichaceae) (*P* < 0.05) and unclassified R4-45B (affiliated to Lentisphaerae) (*P* < 0.05). As was observed in the colon samples, *Dialister* (*P* < 0.05) and *Mitsuokella* (*P* < 0.05), as well as *Prevotella* (affiliated to Prevotellaceae family) (*P* < 0.05) decreased in caecum of the CON-diarrhea pigs. In ileum, only *Paludibacter* (*P* < 0.01) was observed to be higher in relative abundance in CON-diarrhea pigs. However, none of these significant differences was found between diarrhea and non-diarrhea pigs that were fed with RSF.

### Correlations of gut microbiota with host physiology and metadata

#### Histopathology and immunohistochemistry

**Fig 4:**
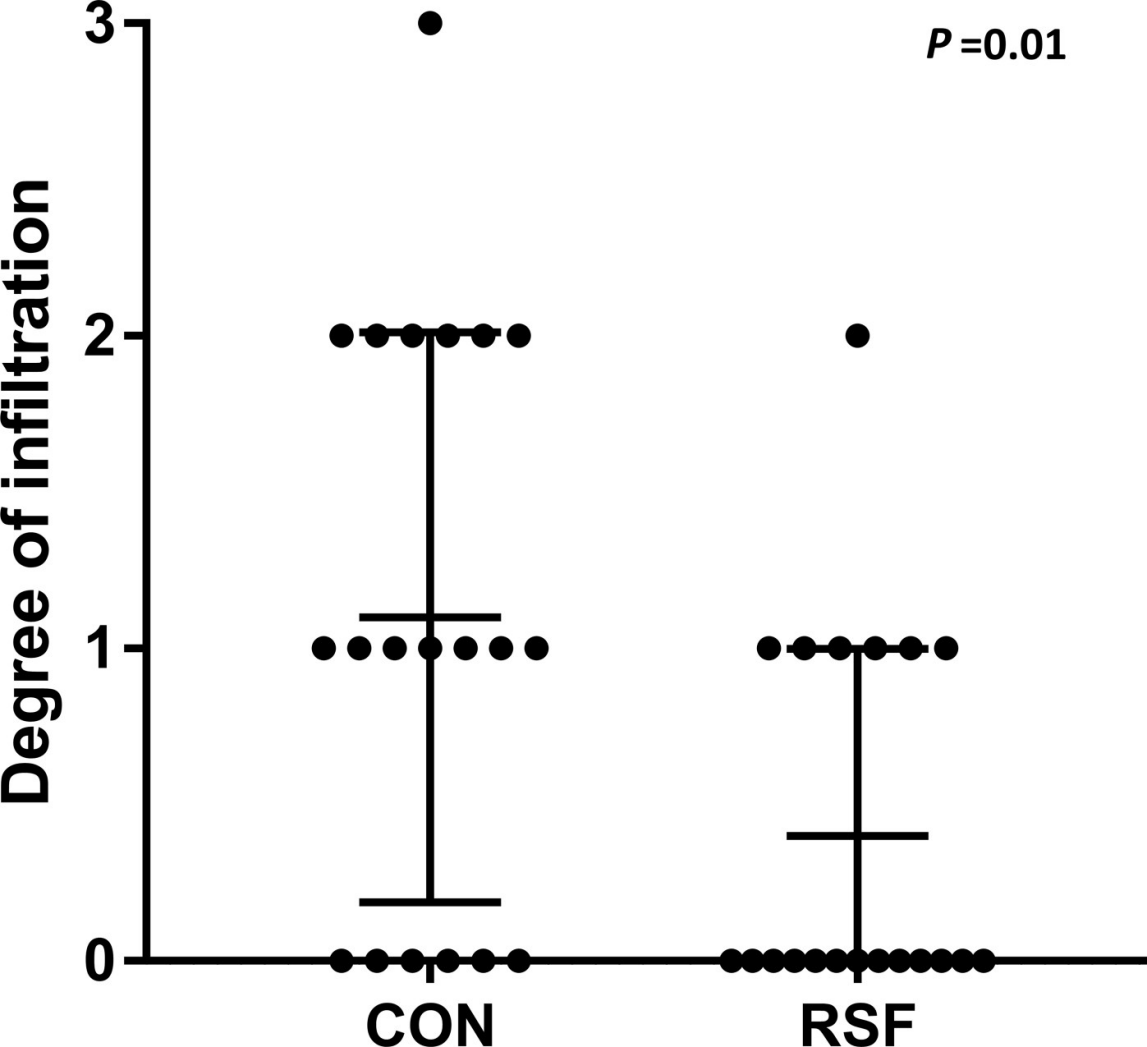
Presence of neutrophils in colon of pigs fed with different diets.

**Fig 5:**
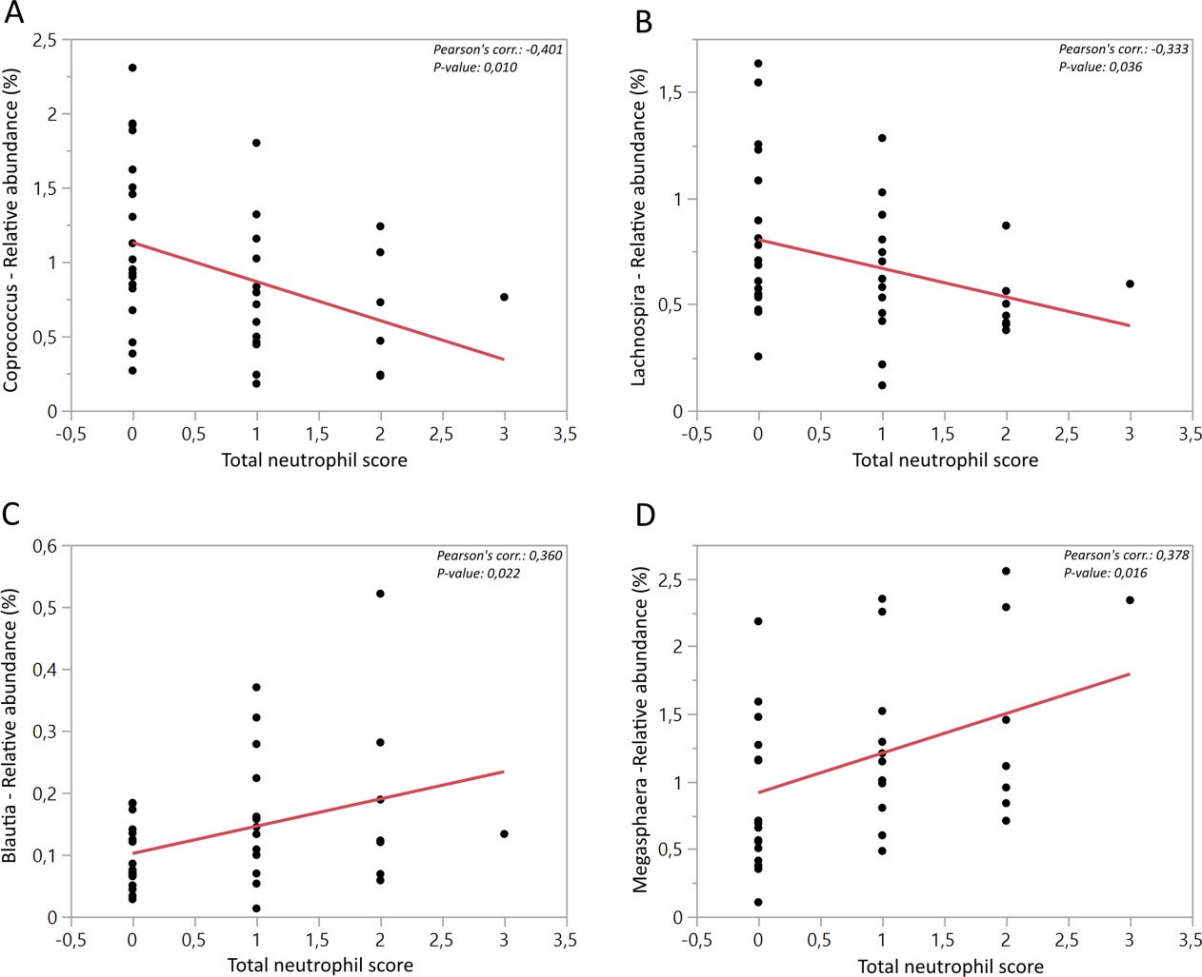
Significant correlations between the bacterial genera and total neutrophil scores in the colon. Graphs A, B, C and D shows the correlations between the semi-quantitative scores of total neutrophils in the colonic mucosa and genera *Coprococcus*, *Lachnospira*, *Blautia* and *Megasphaera* respectively. Pearson correlations and P-values were indicated on top of each graph.

The results of the general histological assessment of internal organs of the pigs were published previously [9], and no differences were observed comparing the two different diets. However, a very mild multi-focal infiltration of neutrophils was observed in the lamina propria of the colon in 21 (8 fed RSF and 13 fed CON) of the 40 animals (Table 2). This mild degree of inflammation tended to be greater in pigs fed CON compared with those fed RSF (*P* = 0.01) (Fig 4). Based on these findings, correlation analysis was conducted between the histopathological parameters and the relative abundance data of bacterial genera. It was noteworthy that negative relationships were found in colon between the presence of neutrophils and the *Lachnospira* (*P* < 0.05) and the *Coprococcus* (*P* = 0.01) genera, which were more abundant in the colon of RSF pigs (Figs 2 and 5). Moreover, *Blautia* (*P* < 0.05) and *Megasphaera* (*P* < 0.05) were positively correlated with the presence of neutrophils.

**Table 2:** Histopathology and immunohistochemistry data for individual pigs.

Pig number	Dietary treatment	*Neutrophil score	Crypt depth (μm)	**Ki67+	Ki67+/Crypt depth	***Number of crypts
1	CON	2	426,7 ± 10,2	24,0 ± 4,9	0,056	3
3	CON	1	536,2 ± 10,5	31,4 ± 1,1	0,058	23
6	CON	2	500,4 ± 14,4	24,8 ± 2,6	0,050	5
7	CON	0	463,6 ± 15,4	12,6 ± 0,9	0,027	5
10	CON	2	528,8 ± 11,2	35,4 ± 1,7	0,067	13
12	CON	0	481,8 ± 8,3	30,7 ± 1,0	0,064	18
14	CON	1	441,1 ± 7,7	18,2 ± 1,2	0,041	12
15	CON	1	468,5 ± 28,2	20,3 ± 2,1	0,043	4
18	CON	3	537,2 ± 26,5	31,5 ± 2,9	0,059	6
19	CON	0	435,7 ± 4,4	27,8 ± 0,9	0,064	36
22	CON	2	614,3 ± 45,3	39,3 ± 2,1	0,064	4
24	CON	1	552,9 ± 12,3	37,4 ± 1,5	0,068	20
26	CON	2	451,7 ± 7,7	26,1 ± 0,9	0,058	20
28	CON	2	589,0 ± 21,7	31,3 ± 4,9	0,053	3
30	CON	0	476,3 ± 7,5	26,3 ± 1,5	0,055	12
32	CON	0	528,7 ± 10,9	23,1 ± 0,5	0,044	10
34	CON	1	490,1 ± 10,6	38,0 ± 1,4	0,077	26
36	CON	0	463,0 ± 11,8	23,3 ± 1,1	0,050	17
38	CON	1	453,9 ± 24,3	38,3 ± 2,8	0,084	15
39	CON	1	489,3 ± 17,5	40,0 ± 2,8	0,082	7
2	RSF	2	523,3 ± 28,4	23,3 ± 2,8	0,045	3
4	RSF	0	566,0 ± 6,8	21,2 ± 1,3	0,037	18
5	RSF	0	535,6 ± 13,8	11,0 ± 0,8	0,021	11
8	RSF	1	466,7 ± 9,7	18,1 ± 0,9	0,039	17
9	RSF	0	418,8 ± 8,0	24,6 ± 1,9	0,059	19
11	RSF	0	463,9 ± 10,6	16,3 ± 0,8	0,035	16
13	RSF	0	451,0 ± 8,7	18,4 ± 3,0	0,041	5
16	RSF	1	499,9 ± 18,0	25,9 ± 1,6	0,052	11
17	RSF	0	480,8 ± 23,2	23,8 ± 3,6	0,049	4
20	RSF	1	405,9 ± 7,3	27,2 ± 1,5	0,067	15
21	RSF	1	455,5 ± 19,2	31,6 ± 1,9	0,069	11
23	RSF	0	521,0 ± 20,3	27,1 ± 1,6	0,052	12
25	RSF	1	465,9 ± 16,2	25,0 ± 1,8	0,054	8
27	RSF	1	459,4 ± 7,9	32,1 ± 1,2	0,070	10
29	RSF	0	-	-	-	-
31	RSF	0	398,5 ± 22,8	38,0 ± 5,0	0,095	4
33	RSF	0	517,7 ± 12,3	28,3 ± 1,6	0,055	7
35	RSF	0	409,0 ± 7,7	31,4 ± 1,4	0,077	14
37	RSF	0	538,2 ± 15,0	27,8 ± 1,5	0,052	12
40	RSF	0	417,2 ± 11,6	29,4 ± 1,6	0,070	13

Crypt depth and Ki67+ data are represented as Mean ± SEM

* Neutrophil scores: 0: none; 1:very mild; 2:mild and 3:moderate

** Ki67+: number of Ki67 positive cells per crypt

*** Number of crypts: the number of the available well-oriented crypts, which were assessed to measure the crypt depth and to count the number of Ki67+ cells.

Mean colonic crypt depth (± SEM) was 473.4 ± 11.6 μm (n = 19) in the RSF group and 496.5 ± 11.5 μm (n = 20) in the CON group (Table 2). Mean number of Ki67+ cells per crypt (± SEM) was 25.3 ± 1.5 (n = 19) in the RSF group and 28.9 ± 1.7 (n = 20) in the CON group. (Table 2); however, there was no statistical significant difference between the dietary groups.

#### Digestibility

In both ileum and colon, bacterial phylotypes that correlate with AID and ATTD of ADF, NDF, CP, DM and GE were different in the RSF and the CON pigs (S1 Table). In ileum of the RSF pigs, some bacteria were positively correlated with AID of both ADF and NDF, and many phylotypes were negatively correlated with the AID of crude protein (CP) compared with the CON pigs. In the CON pigs, many phylotypes in the ileum positively correlated with AID of NDF. Overall, AID and ATTD of the CON and the RSF pigs were correlated with different numbers of phylotypes and different types of the bacterial phylotypes in ileum and colon. These differences suggest that the microbiota is a factor influencing the digestibility of a diet.

#### Metabolites

**Fig 6:**
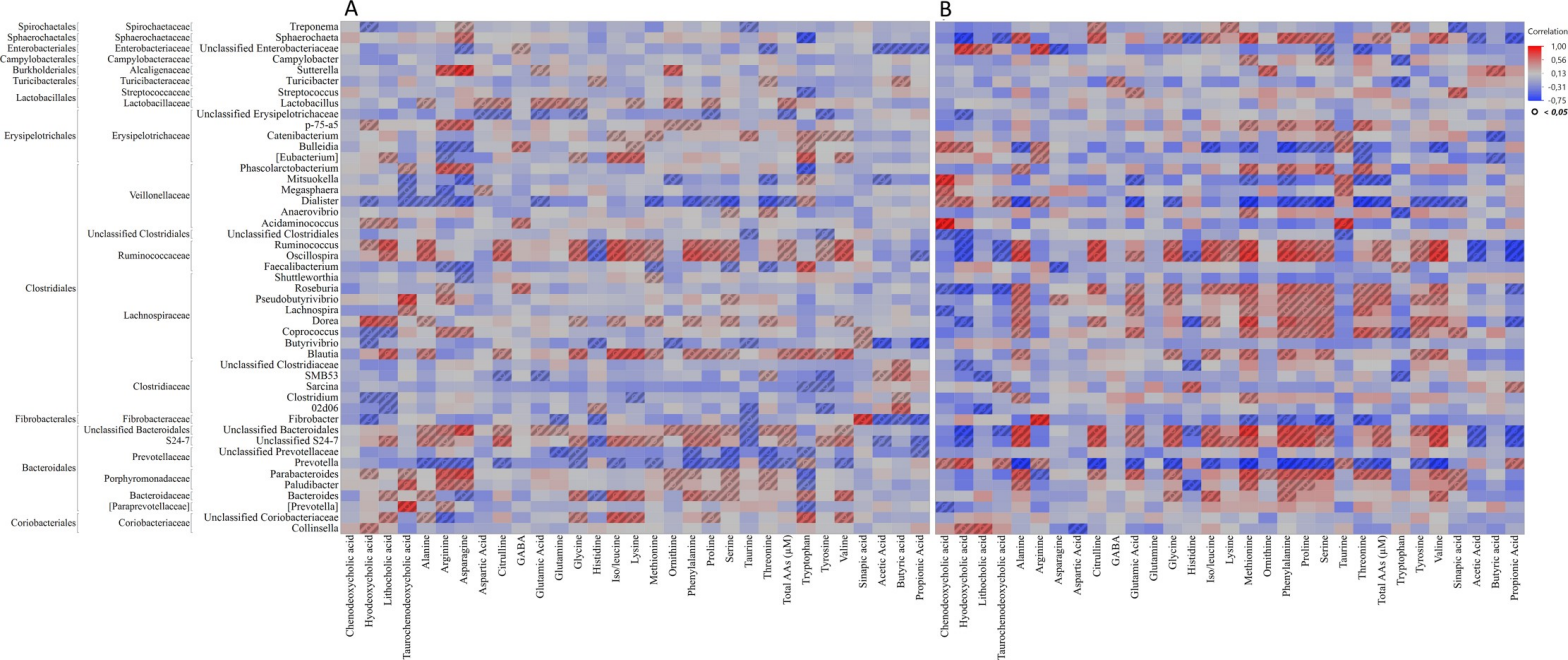
Correlation heatmaps of bacterial genera and metabolites in colon. **(A)** Metabolites and bacteria correlations in the colon of CON diet fed pigs. **(B)** Metabolites and bacteria correlations in the colon of RSF diet fed pigs. The color gradient between red and blue indicates the degree of Pearson’s correlation and the highlighted squares demonstrate the significant correlations (*P-value* < 0.05).

The microbial metabolites such as short-chain fatty acids and secondary bile acids were not found to be affected by the RSF diet, despite of its higher fiber content compared with the CON diet. A correlation analysis was performed between the metabolites and gut microbiota relative abundance data at genus level. Overall, the correlation of the microbiota of the RSF pigs with free amino acids (FAA) was stronger in both ileum and colon compared with the CON pigs (Fig 6 and S1 Fig). The correlations mostly varied for the particular genera in the pigs fed with different diets, for example, the correlations of *Turicibacter* and *SMB53* (affiliated to Clostridiaceae) with most of the FAA in the ileum of CON pigs decreased in the RSF pigs. In the ileum, citrulline, ornithine, taurine and threonine were correlated with most of the ileal bacteria in both the CON and the RSF diets. In addition, there was a positive relationship between aspartic acid, lysine and proline and many of the genera in the ileum of the RSF pigs.

In colon, there were different patterns of correlation of FAAs with the bacteria in the CON and RSF pigs, and stronger correlations between metabolites and bacteria were observed in RSF pigs. The genera affiliated to the Bacteroidales order, and the Ruminococcaceae and the Lachnospiraceae families, with some exceptions, were positively correlated with most of the FAAs in colon of the RSF pigs (Fig 6). In CON pigs, however, a few of these genera, such as *Ruminococcus*, *Oscillospira*, *Blautia*, Unclassified Bacteroidales, Unclassified *S24-7* and *Parabacteroides*, appeared to be associated with the levels of FAA.

Most of the bacteria in the ileum were positively correlated with the levels of propionic acid regardless of the dietary treatment, and negatively correlated with butyric acid in the CON pigs. Moreover, the associations between bacteria and bile salts were also different between the dietary treatments. Most remarkably, the ileal microbiota of RSF pigs, particularly phylotypes of Bacteroidales and some phylotypes of Clostridiales, were positively correlated with lithocholic acid in the RSF pigs, while negative associations were found between the ileal bacterial groups and hyodeoxycholic acid in CON pigs. In contrast to the ileum, colonic microbiota was mostly negatively correlated with bile acids in the RSF pigs, while positive correlations were observed in the CON pigs.

## Discussion

Gut microbiota plays a key role in the interaction between the diet and the host physiology, and is one of the most important determinants of gut health [20]. In our study, the effect of RSF as an alternative feed ingredient in diets for young pigs was assessed with respect to gut microbiota and its associations with host parameters.

Rapeseed co-products contain a higher amount of dietary fibers compared with SBM, including cellulose, pectins, and arabinogalactans as well as other insoluble polysaccharides and lignin in the hulls [2]. Dietary fibers in pig diets have been associated with varying reduction in nutrient utilization and net energy values, depending on their type [6]. Concordantly, in our study, Pérez de Nanclares et al. [9] found that RSF fed pigs had lower AID and ATTD values for nutrients and energy, compared with CON pigs. On the other hand, inclusion of fibers in pig diets can be beneficial to pigs’ health due to the physiological properties and fermentation of dietary fibers by gut microbiota that may affect the host favourably in several ways, such as maintaining gut health, stimulating growth of beneficial bacteria in the gut, and improving overall well-being of the animals [3,21,22]. Moreover, dietary fibers in pig diet alleviate the detrimental effects of protein fermentation in the gut, such as production of potentially toxic substances (e.g. ammonia, amines, indoles and phenols) and stimulation of growth of potentially pathogenic bacteria [23,24]. Therefore, it is advantageous to support the growth of beneficial bacteria in the gut with different feeding strategies such as inclusion of dietary fibers in the feed. Our results identified such beneficial effects of high-fiber RSF on particular bacterial phylotypes in the large intestine. *C*. *perfringens*, which is an opportunistic pathogenic species, was less abundant in caecum of RSF pigs compared with CON pigs. This reduction in opportunistic pathogenic bacteria was consistent with a previous study, where the change from SBM to RSM in the diet of growing-finishing pigs lowered the abundance of *C*. *perfringens* in caecum [8]. In addition, we have found a depression in the relative abundance of *Parabacteroides*, which include opportunistic pathogenic strains [25]. Furthermore, some beneficial bacterial phylotypes, including SCFA producing bacteria, colon health and immune system associated bacteria, such as *Dialister*, *Lachnospira*, *Coprococcus*, *Bulleidia*, *Shuttleworthia*, Unclassified Erysipelotrichaceae and Unclassified Clostridiales, unclassified Coriobacteriaceae [26–31], were more abundant in colon and caecum of the RSF pigs than in the CON pigs. The few studies on the impact of rapeseed on the gut microbiota show that the dietary fibers in rapeseed co-products, including non-starch polysaccharides such as cellulose, arabinogalactans and pectin [2,5], are fermented by the gut microbiota and help maintain the normal physiological functions and gut health of the host [3]. Pectin has increased the butyrate producing Clostridiales group while reducing some populations affiliated to Bacteroidetes, such as *Bacteroides* and *Parabacteroides* in the caecum of rats [32]. Arabinogalactans enhance beneficial gut bacteria, such as *Lactobacillus* and *Bifidobacteria*, and boost immune system via different mechanisms, one of which is possibly rebalancing of gut microbiota and increasing SCFA production [33,34]. Reduction in specific Bacteroidetes populations and increase in SCFA-producers in our study were in agreement with the findings in these studies. Moreover, two of the genera i.e. *Lachnospira* and *Coprococcus*, were present at a higher abundance in the colon of RSF pigs. These genera are affiliated to the Lachnospiraceae family and grouped in the *Clostridium* cluster *XIVa*. The members of this bacterial cluster populate the areas between the mucosal folds in the large intestine [31]. They potentially contribute to immune homeostasis and have anti-inflammatory effect as they have been shown to induce the accumulation and differentiation of colonic T regulatory cells (Tregs) when colonizing the gut of germ free mice, and they have been found to be less abundant in inflammatory bowel disease (IBD) patients compared with healthy subjects [31,35,36]. This is interesting, because a larger number of pigs from the control group had neutrophilic inflammation in the colon compared with the group fed RSF in this present experiment. Neutrophils contribute to intestinal homeostasis by eliminating pathogens; however, this cell population can also damage host tissue and exacerbate inflammation through the release of toxic granule contents and pro-inflammatory molecules [37]. It was previously reported that some proteins in soybean meal such as glycinin and β-conglycinin cause hypersensitivity, e.g. villus atrophy and inflammation, in the small intestine of newly weaned pigs [38,39]. The findings in the present study suggest that RSF may promote a healthier microflora in the colon with less inflammatory stimuli than the soybean meal based control feed although this needs to be supported by further studies.

Despite of these beneficial changes at low taxonomic level, it is important not to disturb the gut microbiota for the maintenance of normal gut function. In our study, there was no marked effect of RSF on the overall composition and diversity of gut microbiota in any of the ileum, caecum and colon of the pigs compared with the CON diet-fed pigs. The overall structure and function of the microbiota remained largely unaffected by RSF without any disturbance of gut health.

Fiber intake in weaner pigs is especially important to reduce the proliferation of pathogenic *E*. *coli* and incidence of diarrhea [40] because SCFA concentrations in ileum decrease in pigs with diarrhea, and this may favor the growth of pathogenic *E*. *coli* [41]. Interestingly, the metabolomics analysis performed on the present study did not detect any significant increase in SCFA concentrations in the digesta of RSF-fed pigs [18]. However, microbiota profiling found that the bacterial phylotypes reputed to be responsible for dietary fiber fermentation and SCFA production were more abundant in RSF pigs. This inconsistency may be the result of most SCFAs being rapidly taken up by the host after production [42], or alternatively the experimental period of 3 weeks was too short to detect a significant change in the metabolic function of SCFA-producing phylotypes. Moreover, cross-feeding between the gut bacteria is a factor that has a great impact on the utilization of substrates and the final balance of the SCFA production [43]. SCFAs are important for colonocyte energy supply and possibly play a role in maintaining intestinal epithelium homeostasis with effects on epithelial proliferation [44]; however, no significant change in colonic crypt depth or proliferative activity in crypts between RSF-fed pigs and CON pigs was observed. The stability of morphometric and proliferative parameters under the present experimental conditions indicates that colonic epithelial homeostasis is maintained in the presence of an increased abundance of SCFA-producing phylotypes.

We have observed post-weaning diarrhea in some of the pigs from both dietary treatment groups. The gut microbiota of RSF pigs that experienced diarrhea retained a relative abundance of bacteria that was similar to the abundance in pigs that did not experience diarrhea. However, CON pigs that experienced diarrhea showed a change in relative abundances. Some bacterial groups including potentially pathogenic phylotypes, were more abundant such as *Paludibacter*, *Parabacteroides*, *Phascolarctobacterium* and *Sphaerochaeta*, while fiber fermenting and health-related bacteria such as *Dialister*, *Mitsuokella* and *Faecalibacterium* [29,45,46] were in lower abundance compared with the non-diarrhea CON pigs. The dietary fibers have been of interest in previous studies for their preventive effect on common problems such as enteric diseases and post-weaning diarrhea [23]. In this context, our findings suggest a health-promoting effect of RSF on weaner piglets due to its high dietary fiber content affecting the abundance of gut bacteria.

The diet type affected the correlations between gut microbiota and digestibility values in our study. The correlations of different bacterial phylotypes with digestibility in CON- and RSF-fed pigs demonstrate functional redundancy in the gut, which is the substitution of different species for the same role in an ecosystem [47]. The change in relative abundance of some bacterial phylotypes caused by RSF may have affected the interaction between the bacteria and feed components modifying the gut environment and the competition between the bacteria. Associations between the gut microbiota and the nutrient absorption in humans has previously been shown as an indication of the role of gut microbiota in nutrient harvest [48,49]. Therefore, the variations in the nutrient digestibility and the gut microbiota between the diets also indicates that microbiota may affect nutrient absorption and digestibility depending on the diet. However, the degree of contribution of gut microbiota in nutrient absorption merits further investigations. In addition, higher fiber content of the diet (e.g. in RSF compared with CON diet) reduces the digestibility of nutrients in the small intestine such that bulkiness and viscosity of dietary fibers may delay the hydrolysis and absorption of nutrients and allow them to travel further through the gastrointestinal tract and reach the large intestine [50]. Moreover, the plant cell wall matrix of the fibers may act as physical barrier and reduce the availability of the nutrients for digestive enzymes and bacteria [50]. Digestibility varies with the age of the pigs as their digestive system is developing and their gut microbiota becomes more stable with increasing fiber-fermentation capacity [3,51]. Therefore, further detailed studies of the impact of RSF on growing-finishing pigs will be valuable as a follow-up study and form the basis of our ongoing efforts.

Associations between the FAAs and the microbiota were overall less pronounced in ileum than in colon, which can be explained by the lower load of bacteria and their limited activities on degradation of amino acids in ileum compared with the large intestine [52]. The high diversity of bacteria in colon leads to competition between the bacteria for the more efficient use of substrates. In colon, different and stronger correlations were observed between FAAs and bacterial genera in the RSF pigs compared with the CON pigs. This suggests that the higher fiber content of RSF, compared with the CON diet, modifies the gut environment with the change in relative abundances of particular bacterial groups. Furthermore, it has been shown that the use of amino acids for synthesis of bacterial protein increases in the large intestine with high-fiber diets, consequently reducing the digestibility of amino acids, which is consistent with the digestibility results [9], and increasing the fecal amino acid excretion [53].

The gut microbiota is associated with the bile acid pool size and composition in gut, which can vary depending on the diet, as one of the influencing factors [54]. In our study, stronger negative correlations were found between the bile acids and the bacterial genera in the RSF pigs compared with the CON pigs in colon. Previously, no significant difference in bile salt concentrations between the CON and the RSF diets was observed [18]. However, the negative associations between the bacteria and bile salts in colon of the RSF pigs are interesting and should be studied further in terms of health-promoting effects of RSF because secondary bile acids (e.g. deoxycholic acid and lithocholic acid) are mostly tumor-promoting and associated with carcinogenesis in human colon [55].

## Conclusion

In conclusion, RSF enhanced the growth of beneficial bacteria and depressed the abundance of opportunistic pathogenic bacteria in the large intestine without disturbing the main gut microbiota structure and activities. These properties contribute to the maintenance of gut health of the host. Moreover, the relative abundances of bacterial phylotypes did not change in RSF diet fed pigs, while some bacterial groups including potentially pathogenic phylotypes were more abundant in the control fed pigs that experienced diarrhea. This is an important finding to be supported with more comprehensive studies, as post-weaning diarrhea is a common cause of mortality and morbidity in young pigs. These beneficial effects of RSF on gut microbiota have the potential to contribute the growth and health of the pigs and promote RSF as an alternative feed ingredient to soybean. Furthermore, optimization of the amount of rapeseed co-products or their dietary fiber composition in the diet formulation may help to improve rapeseed diets, by minimizing the reduction in nutrient digestibility and maximizing the health-promoting effects on gut microbiota.

## Supporting information

S1 Fig Correlation heatmaps of bacterial genera and metabolites in ileum. (A) Metabolites and bacteria correlations in the ileum of CON diet fed pigs. (B) Metabolites and bacteria correlations in the ileum of RSF diet fed pigs. The color gradient between red and blue indicates the degree of Pearson’s correlation and the highlighted squares demonstrate the significant correlations (P-value < 0.05).
(TIF)

S1 Table Bacteria and digestibility correlations for ileum and colon. (XLSX)

S2 Table The diarrhea incidences for individual pigs. (XLSX)
