# Supplementary figures and images for: Gut microbiota profiling in Norwegian weaner pigs reveals potentially beneficial effects of a high-fiber rapeseed diet
Source: PLoS One. 2018 Dec 20;13(12):e0209439. doi: 10.1371/journal.pone.0209439 (PMC6301702; doi:10.1371/journal.pone.0209439)

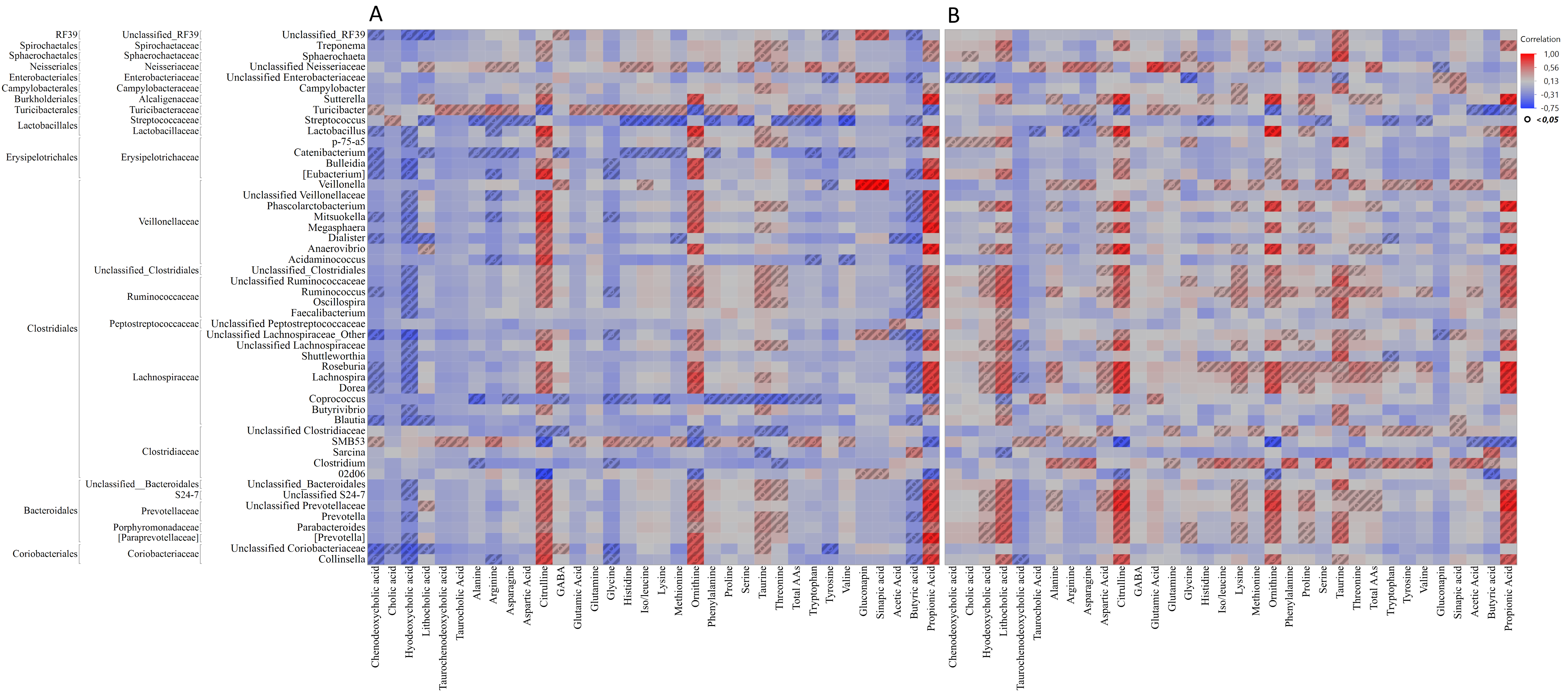

Supplement: S1 Fig — (A) Metabolites and bacteria correlations in the ileum of CON diet fed pigs. (B) Metabolites and bacteria correlations in the ileum of RSF diet fed pigs. The color gradient between red and blue indicates the degree of Pearson’s correlation and the highlighted squares demonstrate the significant correlations (P-value < 0.05). (TIF) [file pone.0209439.s001.tif]
